# Association of COVID-19-imposed lockdown and online searches for toothache in Iran

**DOI:** 10.1186/s12903-021-01428-z

**Published:** 2021-02-15

**Authors:** Ahmad Sofi-Mahmudi, Erfan Shamsoddin, Peyman Ghasemi, Ali Mehrabi Bahar, Mansour Shaban Azad, Ghasem Sadeghi

**Affiliations:** 1 Cochrane Iran Associate Centre, National Institute for Medical Research Development (NIMAD), West Fatemi St., 1419693111 Tehran, Iran; 2 Department of Health Economics, School of Public Health, Tehran University of Medical Sciences, Tehran, Iran; 3 Department of Health Policy and Management, School of Public Health, Tehran University of Medical Sciences, Tehran, Iran; 4 Department of Cardiovascular Surgery, Shahid Chamran Heart Educational, Medical and Research Center, Isfahan University of Medical Sciences, Isfahan, Iran; 5 Bureau of Dentistry, Vice Chancellery for Treatment, Ministry of Health and Medical Education, Tehran, Iran

**Keywords:** COVID-19, Google trends, Iran, Toothache

## Abstract

**Background:**

Novel coronavirus disease-2019 (COVID-19) has impacted populations in many ways worldwide, including access to oral health services. This study aims to assess the association between lockdown due to COVID-19 and online searches for toothache in Iran using Google Trends (GT).

**Methods:**

We investigated GT online searches for toothache within the past five years. The time frame for data gathering was considered as the initiation and end dates of the national lockdown in Iran. We performed one-way ANOVA statistical test to compare relative search volumes (RSVs) between the year 2020 and 2016–2019 for the whole country. Then we investigated the possible association of RSVs in provinces with dentists’ density, prevalence of current daily smokers, Human Development Index (HDI), Internet access, and fluoride concentration in water using linear regression.

**Results:**

When comparing 2020 with the previous four years, there was a rise of 2020 RSVs versus all previous years combined and each year (P < 0.001 for all of them). In the linear model for the year 2020, HDI (B = − 3.29, 95% CI: (− 5.80, − 0.78), P = 0.012) had a strong negative relationship with provincial RSVs. HDI mostly had strong positive relationship with provincial RSVs in prior years. Fluoride concentration (B = − 0.13, 95% CI: (− 0.24, − 0.03), P = 0.017) and dentists’ density (B = − 0.04, 95% CI: (− 0.25, 0.17), P = 0.669) were also negatively associated with RSVs in 2020. These associations were mostly negative in the previous years as well. Internet access (B = 0.36, 95% CI: (− 0.38, 1.09), P = 0.325) and prevalence of daily smokers (B = 0.33, 95% CI: (0.13, 0.53), P = 0.002) were positively associated with RSVs.

**Conclusion:**

The RSVs for toothache in 2020 have increased due to COVID-19-imposed lockdown compared with the same period in the past four years. This increase was related to socioeconomic factors.

**Supplementary Information:**

The online version contains supplementary material available at 10.1186/s12903-021-01428-z.

## Background

While the outbreak of novel coronavirus disease-2019 (COVID-19) has afflicted the populations in many aspects globally, noticing its implicit consequences is of utmost importance. This can include socioeconomic, political, cultural, or even clinical aspects which could harm equitable access to healthcare during public health emergencies [1]. Providing the best possible clinical care could be mentioned as a critical duty for healthcare systems in each country or region in the era of COVID-19 [2–4]. Setting urgent medical centres to provide necessary medical services for patients during the outbreak is a practised experience for many countries [3, 5, 6]. Given that more hassles (e.g. patient overload, shortage of personal protective equipment, etc.) can rise during a public health emergency, the need for urgent healthcare can be of a more vivid nature [7–9].

Meanwhile, the accessibility of online resources (media, social networks, scientific websites) is constantly increasing, and the way the general population seeks information is parallelly changing [10]. Online searches can serve as a surrogate measure of disease awareness in public settings, thereby necessitating the need for monitoring online care seeking behaviour (CSB) of the general population [11]. Assessing CSB online tools has been a topic of interest in the previous years, especially during the COVID-19 pandemic [12, 13]. While knowing the critical impacts of oral health status on the daily life of every individual, people have been increasingly searching about oral diseases [14–16]. The way people seek information via Google using various keywords is an implicit exhibition of their online CSB. Google Trends (GT) has already been proven to be a valid and reliable tool for assessing the online search trends of populations in the medical field [17, 18].

Pain is considered to be one of the most important chief complaints in dental patients globally [19, 20]. Accordingly, addressing patients’ needs for dental services when they are in pain (emergency treatments) should be critically concerned during the COVID-19 outbreak. Carter et al. have reported the acute pulpitis and periapical diseases as the most common complaints of adult and pediatric patients in urgent dental care centres of Newcastle Dental Hospital during COVID-19 [6]. Another study claimed the irreversible pulpitis and dental trauma to be the most frequent diagnoses in pediatric patients in an Urgent Dental Care Centre in the North East of England and North Cumbria [21].

Iran is no exception to this. The need for urgent dental services in the country is further reinforced by the presence of a poor to fair oral health status of the general population and especially among 35- to 44-year-old Iranians [22]. One round of national and several occasions of state-wide lockdowns have been issued in Iran as counteracts to COVID-19 spread which could have caused some challenges for each individual to receive the dental healthcare they needed during the lockdown [23]. Despite some reports about the reopening of dental offices during the COVID-19 pandemic in Iran, dental offices were generally closed during the lockdown [24, 25]. Having detailed information about the general CSB could help in addressing the public need for dental healthcare and effective health service planning during a public health emergency in the future. Accordingly, we aimed to assess the association between lockdown due to COVID-19 and searches for toothache using GT in Iran. Our research questions were as follows:

**RQ1**: Whether RSVs for toothache during the 2020 COVID-19 lockdown have increased compared with the same period in the past four years in Iran?

**RQ2**: Whether the province-level RSVs were associated with dentists’ density (per 100,000), prevalence of current daily smokers, Human Development Index (HDI), Internet access, and fluoride concentration in water?

## Materials and methods

### Google Trends

In this cross-sectional study, we used GT to investigate internet search activity during the lockdown due to COVID-19 epidemic in Iran. This web service determines the proportion of searches for a user-specified term in all the searches performed on Google Search engine. It then provides an RSV, which is the query share of a particular term for a given location and period, normalised by the highest query share of that term over the time-series and presented on a scale from 0 to 100 [26]. RSV is presented as “Interest” value in GT website.

As Persian is the official language in Iran, we used “دندان درد” (as search term) in GT, meaning “toothache” in formal Persian. There is another word for toothache in informal Persian, “دندون درد”; however, since the formal term is used more widely in Iran, we only collected its pertaining RSVs.

Then, we gathered provincial RSVs’ data from the start of the lockdown in Iran (2020-03-14) till 3 months (12 weeks) afterwards (2020–06-14). We did the same for a similar period in 2016–2019 and chose the past year (2019) as the control event. The total number of provinces in Iran is 31.

### Statistical analysis

We plotted the trend of “دندان درد” from 5 years ago to date. We performed one-way ANOVA statistical test to identify whether there is a statistical difference for RSV scores between the year 2020 and 2016–2019 for the whole country after normalising the data based on the Internet penetration rate. Then we investigated the possible association of RSVs in provinces with some parameters that are important for health services plannings including: dentists’ density in 2019 (per 100,000) (retrieved from Iran’s Ministry of Health data), the prevalence of current daily smokers in 2016 (retrieved from Iran’s STEPs 2016 study [27], vizit.report/panel/steps/en/main.html), Human Development Index (HDI) in 2016 [28], Internet access in 2019 (retrieved from Iran’s Ministry of Information and Communications Technology, mis.ito.gov.ir/ictindex/), and fluoride concentration in water in 2015 [29] with linear regression. Statistical analyses were done using Python v3.6.7 (2018–10-20) (Python Software Foundation, Delaware, United States. http://www.python.org) on Google Colab. No statistically significant level was considered according to the latest recommendations of the American Statistical Association [30].

## Results

**Fig. 1:**
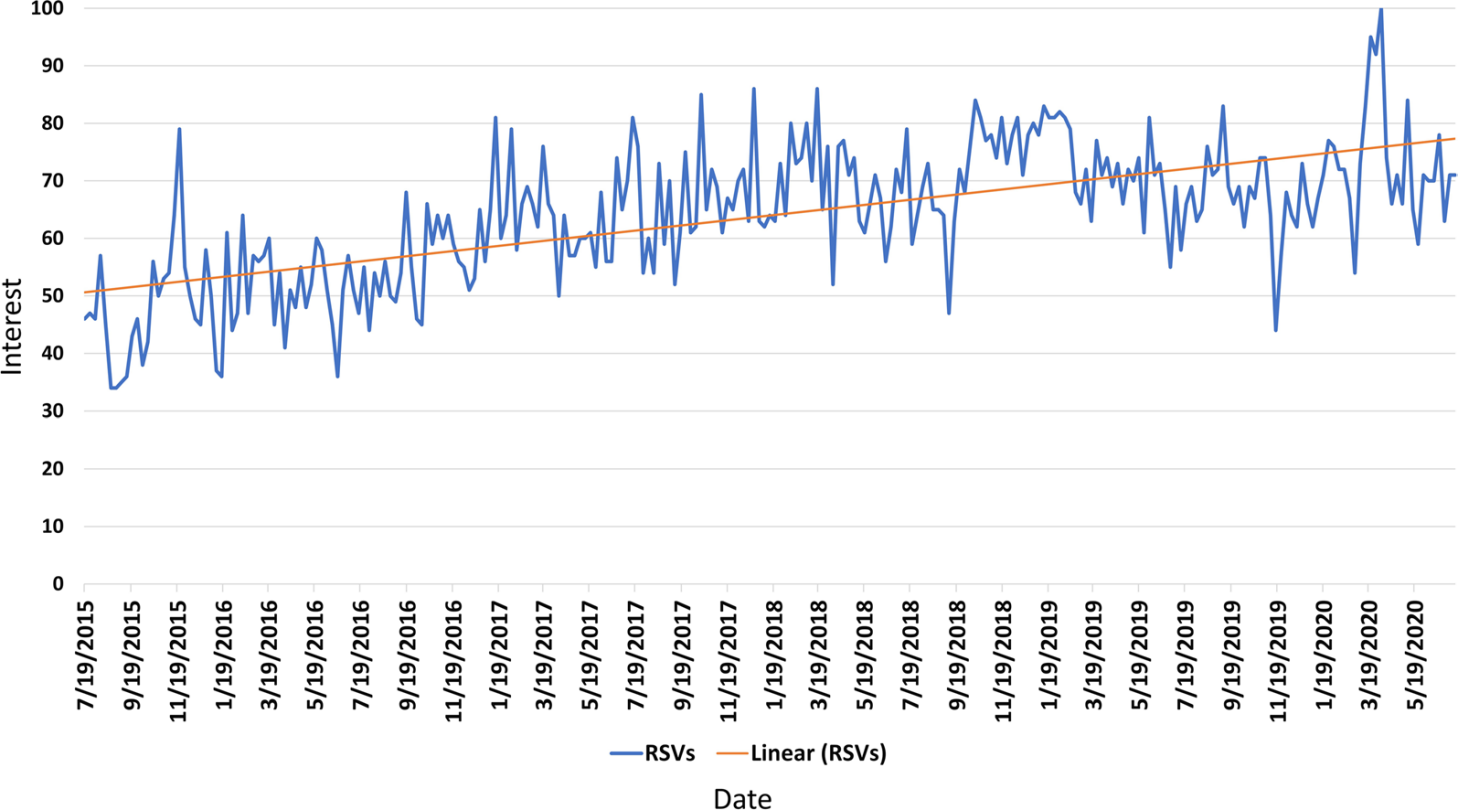
Relative search volume’s trend for the term “toothache” in Iran in the past five years

**Fig. 2:**
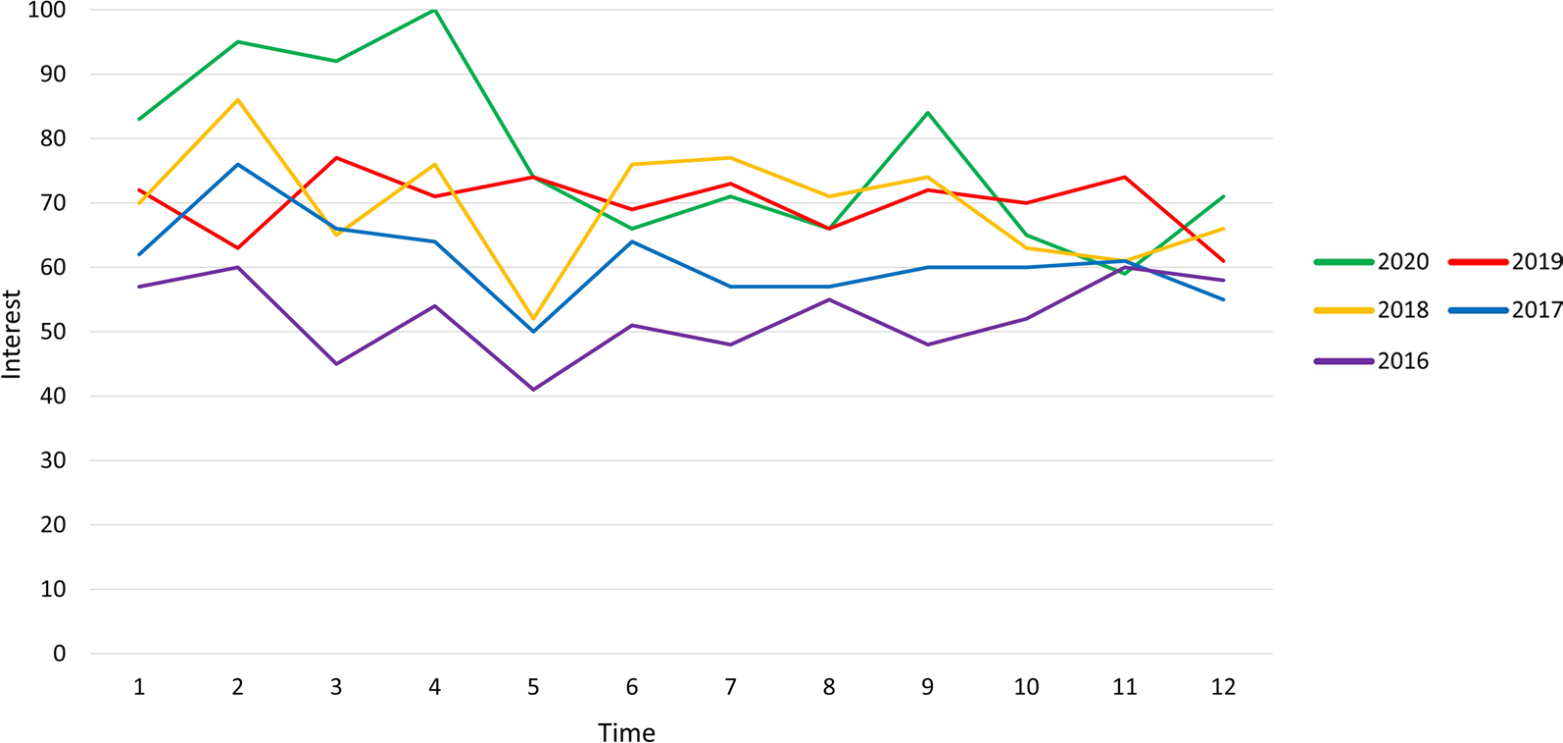
Comparing the RSVs for 2020 until three months after the lockdown with similar weeks in the past four years

**Fig. 3:**
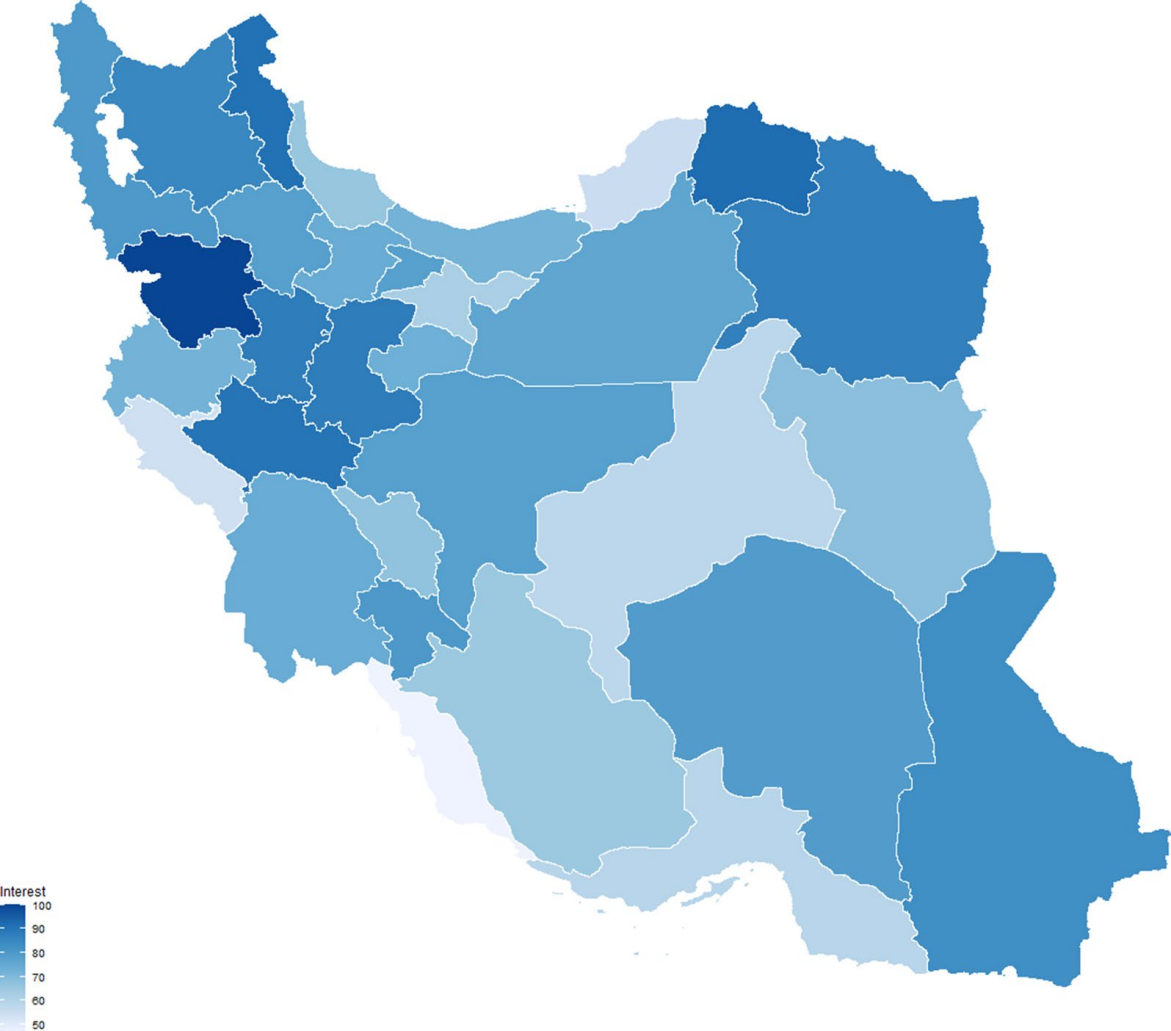
Relative search volumes for the term “toothache” in the provinces of Iran in 2020 (created using ggplot 3.3.0 (ggplot2.tidyverse.org) on R 3.6.0 (R Core Team, 2019, r-project.org)

Overall, the RSVs trend was increasing in the past five years (Fig. 1). The trend of RSVs for 2020 was increasing to a peak till the fourth week of the lockdown, then decreasing (Fig. 2). When comparing 2020 with the previous four years (combined and indiviually), there was a rise in 2020 RSVs (P < 0.001 for all of them). Figure 3 shows the RSVs for 2020 in the provinces of Iran (maps for the other years are illustrated in Additional file 1).

In the linear model for the year 2020, HDI (B = − 3.29, 95% CI: (− 5.80, − 0.78), P = 0.012) had a strong negative relationship with provincial RSVs. This assoication was mostly positive in prior years. Fluoride concentration (B = − 0.13, 95% CI: (− 0.24, − 0.03), P = 0.017), and dentists’ density (B = − 0.04, 95% CI: (− 0.25, 0.17), P = 0.669) were also negatively associated with RSVs in 2020. These associations were mostly negative in the previous years as well. Internet access (B = 0.36, 95% CI: (− 0.38, 1.09), P = 0.325) was positively associated with RSVs whereas in prior years it had a negative relationship with RSVs. Prevalence of daily smokers (B = 0.33, 95% CI: (0.13, 0.53), P = 0.002) was also positively associated with RSVs. Full details of the linear models for each year are shown in Table 1.

**Table 1:** Linear regression results

	2020	2019	2018	2017	2016
(Constant)	0.69*** (0.33, 1.05)	− 0.41 (− 1.05, 0.23)	0.05 (− 0.55, 0.65)	0.33 (− 0.29, 0.95)	0.002 (− 0.55, 0.55)
Human Development Index (HDI)	− 3.29** (− 5.80, − 0.78 (	4.30 (− 0.19, 8.78)	1.56 (− 2.65, 5.78)	− 0.69 (− 5.04, 3.67)	1.83 (− 2.02, 5.69)
Internet access	0.36 (− 0.38, 1.09)	− 0.59 (− 1.90, 0.72)	− 0.50 (− 1.73, 0.74)	− 0.22 (− 1.49, 1.06)	− 1.13* (− 2.26, 0.00)
Fluoride concentration	− 0.13* (− 0.24, − 0.03)	− 0.12 (− 0.31, 0.07)	− 0.16 (− 0.33, 0.02)	− 0.09 (− 0.27, 0.09)	0.08 (− 0.08, 0.24)
Dentists’ density	− 0.04 (− 0.25, 0.17)	− 0.34 (− 0.72, 0.03)	− 0.074 (− 0.43, 0.28)	0.14 (− 0.23, 0.50)	− 0.01 (− 0.33, 0.31)
Daily smokers	0.33** (0.13, 0.53)	− 0.07 (− 0.42, 0.29)	− 0.17 (− 0.50, 0.16)	0.02 (− 0.32, 0.36)	0.20 (− 0.10, 0.50)
R^2^	0.541	0.274	0.234	0.092	0.320

95% compatibility intervals in brackets (*p < 0.05; **p < 0.01; ***p < 0.001)

## Discussion

We found that RSV for toothache in Iran has increased during COVID-19-imposed lockdown compared with the same period in the past four years. When analysing RSVs for provinces, we realised that HDI had a strong negative association with RSVs in 2020. Of other assessed factors, fluoride concentration and dentists’ density were negatively associated with 2020 RSVs whereas Internet access and prevalence of daily smokers had positive associations.

While the prevalence of permanent teeth caries in Iran has reached a plateau in the recent years [14], searching for toothache has increased. One probable contributing factor to this rise might be the continuously growing Persian content on the web. To have a better view of the situation, when we compare the Persian Wikipedia in 2016 with 2020, we will see the number of articles has increased 1.5 times [31]. With this outburst, it is plausible for each individual to seek the needed information via the available online resources, thereby erecting the main question of our reseach.

Though the national COVID-19 lockdown mostly overlapped with the national holiday of “Nowruz” in Iran (almost lasts 15 days after 20th March, every year), it seems that the lockdown affected people’s CSB more markedly in 2020. This may be due to the compulsory closure of all dental offices during the lockdown; an unprecedented event as some dental offices were open during the holidays in the previous years. Supposing that the fear of contracting COVID-19 in dental offices prevented some from attending dental offices, it could be a contributing factor to observing a rise in RSVs during the 2020 lockdown in Iran.

Being broadly known that oral diseases, especially dental caries, are related to socioeconomic factors (31), and deprived populations are generally worse off in terms of oral health status compared to populations with better socioeconomic status, it was plausible to find that more developed regions in Iran would exhibit lower RSVs and vice versa. This was substantiated by our results as provinces with lower HDI had higher RSVs for toothache. We found a strong negative association between HDI and searches for toothache (B = − 3.29, P = 0.012), which was not affected by the Internet penetration rate (B = 0.36, P = 0.325) in each province.

Provinces with higher fluoride concentrations showed lower RSVs during the lockdown in 2020. This negative association was observed in 2019 as well. There is a body of literature that water fluoridation is a cost-effective community-based preventive approach to manage dental caries [32, 33]. However, currently, there is no water fluoridation programme in Iran, and the amount of fluoride in drinking water is different among various provinces based on the characteristics of the natural water. Southern provinces such as Bushehr have higher fluoride concentrations in the drinking water [29], and lower dental caries experience [34]. All of these are in line with lower search interests for toothache in regions with higher fluoride concentrations.

It is believed that smoking is associated with several health conditions, including oral cancers [35], periodontal diseases [36], and dental caries [37]. This study found a positive association between smoking and provincal RSVs for toothache, which could mean that provinces with higher number of current smokers—and consequntly higher rates of associated dental diseases—may had higher figures of individuals who experienced toothache during the lockdown [37].

A notable finding of this study was that the dentists’ density and Internet penetration rate were not strongly associated with RSVs in 2020. In recent years, the number of dental schools and dentists in Iran has been increasing dramatically [38]. Be that as it may, there are some doubts about the effectiveness of such increases in promoting public oral health status [39].

## Limitations

Our findings should be interpreted with caution. Whilst Google shows the most market share among online search engines in Iran (99.11% as of November 2020) [40], this study only assessed the CSB among Google users. Furthermore, although the Internet penetration rate is above 50% in all provinces [41], current results may not represent all of the demographics in general population. Another issue was that we could not have access to the raw data on GT; accordingly, we do not know about the exact times that a single person has searched about toothache. This could lead to a probable error of duplication in the records (online searches). Additionally, we did not have access to up-to-date data on the prevalence of current daily smokers, Human Development Index (HDI), and fluoride concentration in water, leading to a confounding bias [42].

## Conclusion

The extensive afflictions of COVID-19 shades over the field of dentistry. Care seeking behaviour of the Iranians concerning toothache, using Google search engine, shows an increase in 2020 compared with the previous four years. While investigating, we found that HDI, fluoride concentration in the regional water and dentists’ density were negatively associated with RSVs among the provinces. This relation was positive between prevalence of daily smokers and RSVs in 2020. These findings can implicitly show the importance of different health-planning policies in Iran being mirrored in general populations’ online CSB, and especially during COVID-19.

## Supplementary Information

**Additional file 1:** Relative search volumes in the provinces of Iran from 2016 to 2019.

## Data Availability

The datasets generated during the current study are available in the figshare repository, https://doi.org/10.6084/m9.figshare.13108070.

## Abbreviations

COVID-19: Coronavirus disease-2019
CSB: Care seeking behaviour
GT: Google Trends
RSV: Relative search volume
HDI: Human Development Index
