# Supplementary material for: Association of COVID-19-imposed lockdown and online searches for toothache in Iran
Source: BMC Oral Health. 2021 Feb 15;21:69. doi: 10.1186/s12903-021-01428-z (PMC7883957; doi:10.1186/s12903-021-01428-z)

Association of COVID-19-imposed lockdowns and online searches for toothache in Iran

Ahmad Sofi-Mahmudi^1^, DDS, Erfan Shamsoddin^1*^, DDS, Peyman Ghasemi^2^, MSc, Ali Mehrabi Bahar^3^, MD, Mansour Shaban Azad^4^, MD, Ghasem Sadeghi^5^, DDS

^1^ Cochrane Iran Associate Centre, National Institute for Medical Research Development (NIMAD), Tehran, Iran.

^2^ Department of Health Economics, School of Public Health, Tehran University of Medical Sciences, Tehran, Iran.

^3^ Department of Health Policy and Management, School of Public Health, Tehran University of Medical Sciences, Tehran, Iran.

^4^ Department of Cardiovascular Surgery, Shahid Chamran Heart Educational, Medical and Research Center, Isfahan University of Medical Sciences, Isfahan, Iran.

^5^ Bureau of Dentistry, Vice Chancellery for Treatment, Ministry of Health and Education, Tehran, Iran.

# Appendices

Appendix 1. RSVs for provinces of Iran in 2016-2019


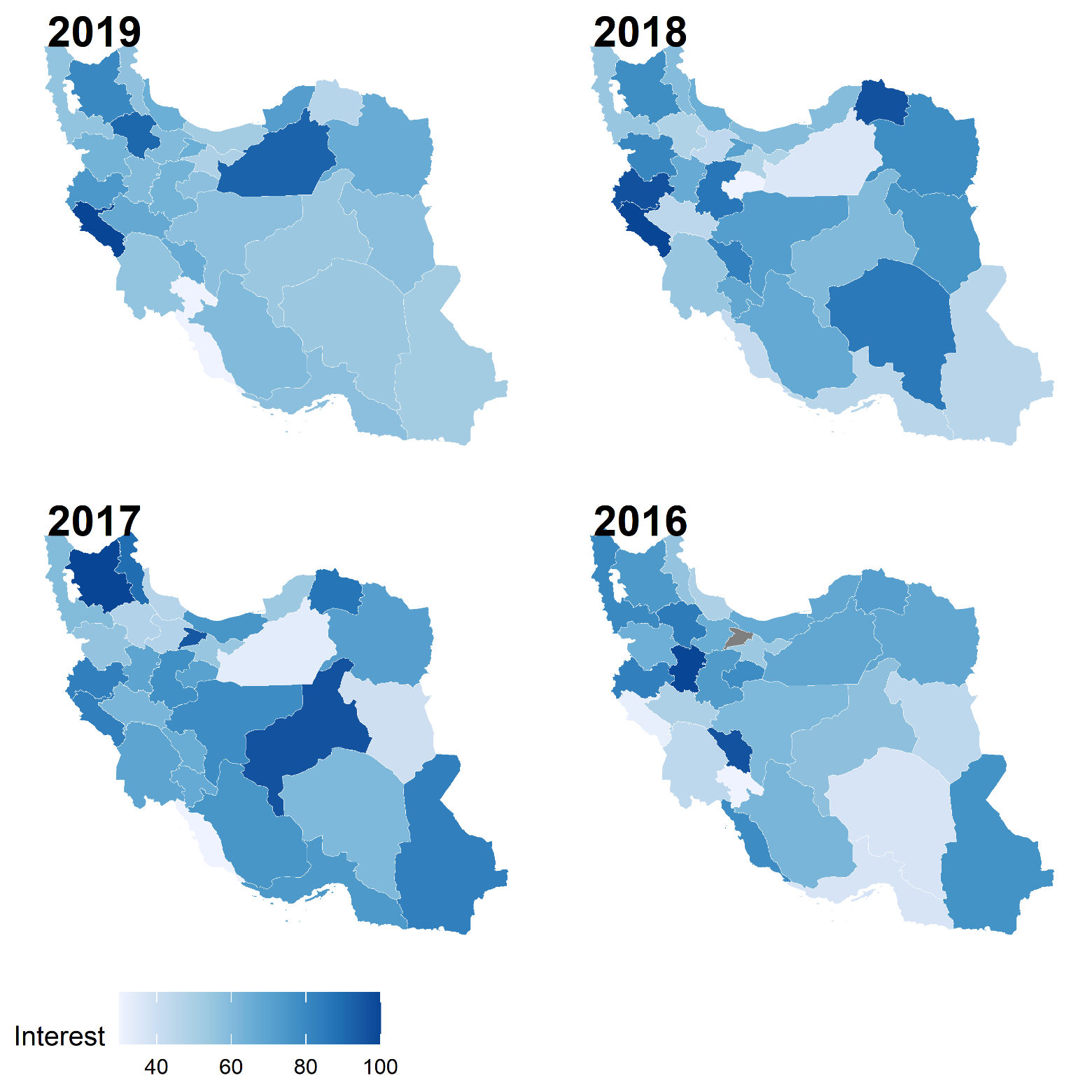

Supplement: Supplementary file 1 — Additional file 1: Relative search volumes in the provinces of Iran from 2016 to 2019. [file 12903_2021_1428_MOESM1_ESM.docx]
